# Characterization of a Novel Group of *Listeria* Phages That Target Serotype 4b *Listeria monocytogenes*

**DOI:** 10.3390/v13040671

**Published:** 2021-04-14

**Authors:** Yaxiong Song, Tracey L. Peters, Daniel W. Bryan, Lauren K. Hudson, Thomas G. Denes

**Affiliations:** Department of Food Science, University of Tennessee, Knoxville, TN 37996, USA; ysong35@vols.utk.edu (Y.S.); tpeter21@vols.utk.edu (T.L.P.); dbryan8@utk.edu (D.W.B.); lkhudson@utk.edu (L.K.H.)

**Keywords:** *Listeria monocytogenes*, bacteriophage, phage, serotype 4b

## Abstract

*Listeria monocytogenes* serotype 4b strains are the most prevalent clinical isolates and are widely found in food processing environments. Bacteriophages are natural viral predators of bacteria and are a promising biocontrol agent for *L. monocytogenes*. The aims of this study were to characterize phages that specifically infect serotype 4b strains and to assess their ability to inhibit the growth of serotype 4b strains. Out of 120 wild *Listeria* phages, nine phages were selected based on their strong lytic activity against the model serotype 4b strain F2365. These nine phages can be divided into two groups based on their morphological characteristics and host range. Comparison to previously characterized phage genomes revealed one of these groups qualifies to be defined as a novel species. Phages LP-020, LP-027, and LP-094 were selected as representatives of these two groups of phages for further characterization through one-step growth curve and inhibition of serotype 4b *L. monocytogenes* experiments. *Listeria* phages that target serotype 4b showed an inhibitory effect on the growth of F2365 and other serotype 4 strains and may be useful for biocontrol of *L.monocytogenes* in food processing environments.

## 1. Introduction

*Listeria monocytogenes* is a Gram-positive foodborne pathogen that infects humans and animals [1]. *L. monocytogenes* is widely isolated from soil, agriculture environments, and urban environments, and can tolerate high salt concentrations and a broad range of temperatures and pH levels [2,3,4,5]. Contamination of the food processing environment with *L. monocytogenes* can lead to consumers ingesting the pathogen, which can cause the potentially fatal invasive disease listeriosis [6]. The global burden of listeriosis cases has been estimated at 23,150 annually, with a mortality rate of 26% [7]. Within the United States alone, there is an estimated 2518 annual cases, with a 20% mortality rate [8,9], and economic losses caused by listeriosis were more than $3.1 billion in 2018 [10].

Bacteriophages are natural viral predators of bacteria, which infect and lyse specific host strains [11]. High specificity, self-replication capability, and tolerance of a wide range of temperatures and pHs [12] make bacteriophages a promising candidate for biocontrol of *L. monocytogenes* in the food processing environment [13]. *Listeria* phage biocontrol products have been approved for use by the United States Food and Drug Administration since 2006. These products are marketed to control *L. monocytogenes* contamination on food and in food processing plants. Currently used *Listeria* phages have been characterized as belonging to the genus *Pecentumvirus* [14]. *Pecentumvirus* phages have been shown to utilize rhamnose and N-acetylglucosamine of wall teichoic acids as binding receptors during the adsorption step of infection [15]. Presence or absence of these sugars corresponds to the various serotypes of *L. monocytogenes* [16]; thus, *Listeria* phages show some level of serotype specificity [17].

Based on cell surface antigenic determinants, *L. monocytogenes* can be divided into at least 13 serotypes. Serotype 4b strains account for most clinal isolates from humans, causing about 50% of illnesses; serotype 1/2a ranks second and is associated with 27% of cases [18,19,20,21]. Serotype 1/2a and 4b are the most frequently recovered from food and environmental samples [20,22,23,24]. Previous phage host-range studies employing efficiency of plaquing assays showed three *Pecentumvirus* phages, LP-048, LP-125, and A511, that effectively form plaques against serotype 1/2 strains and serotype 4b strain F2365 [25]; however, *Homburgvirus* LP-018, which also shows some potential use in biocontrol applications [26], was not able to infect this model strain [25]. To increase the diversity of characterized phages available for use against serotype 4 strains, we screened and characterized nine *Listeria* phages that show strong infectivity against the serotype 4b *L. monocytogenes* strain F2365.

## 2. Materials and Methods

### 2.1. Bacterial Strains and Bacteriophages

All bacterial strains in this study are listed in Table 1. *L. monocytogenes* MACK was used for phage titering and phage propagation of *Listeria* phages A511, LP-048, and LP-125. *L. monocytogenes* F2365 is the *L. monocytogenes* serotype 4b standard strain that was used for phage titering and phage propagation of the remaining *Listeria* phages (LP-020, LP-021, LP-024, LP-027, LP-053, LP-054, LP-057, LP-085, and LP-094). *L. monocytogenes* 10403S is a *L. monocytogenes* serotype 1/2a model strain. FSL D4-0014 and FSL D4-0119 are mutants of *L. monocytogenes* 10403S that lack N-acetyl glucosamine and rhamnose in their wall teichoic acid, respectively. All the strains were stored at −80 °C in Brain Heart Infusion (BHI) supplemented with 15% (*w*/*v*) glycerol and grown on 1.5% (*w*/*v*) BHI agar plates at 37 °C. Overnight cultures for each strain were inoculated with a single colony from a streak plate into BHI broth and grown at 37 °C in a shaking water bath at 160 RPM.

All *Listeria* phages in this study are listed in Table 2. *Listeria* phages LP-048 and LP-125 were well-studied phages that are able to infect serotype 1/2a strains. *Listeria* phage A511 is a broad range phage that is able to infect both serotype 1/2a strains and 4b strains [27,28]. The other *Listeria* phages (LP-020, LP-021, LP-024, LP-027, LP-053, LP-054, LP-057, LP-085, and LP-094) were included in the study due to their ability to show strong lytic activity against *L. monocytogenes* F2365. All phages were titered on lysogeny broth morpholino-propane sulfonic acid (LB-MOPS) agar supplemented with 0.1% glucose, 1 mM CaCl_2_, and 1 mM MgCl_2_ by 10 µL spot assay and were incubated at 25 °C overnight (16 ± 2 h). Phage stocks were prepared by liquid amplification. A culture of the host strain was grown to an OD_600nm_ of 0.2, infected with the phage at a multiplicity of infection (MOI) of 0.1; after 3 h of incubation at 25 °C in a shaking water bath, the infected culture was filtered with a 0.45 µm SCFA sterile filter, and then centrifuged at 12,000× *g* at 4 °C for 2 h. The supernatant was then removed, and the pellet was resuspended in SM buffer (0.1% *v*/*v* gelatin, 0.05 M Tris-Cl pH 7.5, 0.58% *w*/*v* NaCl, 0.2% *w*/*v* MgSO_4_·7H_2_O) by static incubation at 4 °C for 24 h, then filtered with a 0.20 µm SCFA sterile filter and transferred to a sterile tube as new phage stock. All phage stocks were stored at 4 °C in SM buffer. Storage at 4 °C in liquid media with structurally similar phages has been demonstrated to maintain stable titers for months to years with minimal degradation of stock viability [29,30,31].

**Table 1:** *Listeria monocytogenes* strains.

Strain	Serotype	Reference or Original
10403S	1/2a	Bishop and Hinrichs, 1987 [32]
MACK	1/2a	Hodgson, 2000 [33]
F2365	4b	Nelson, 2004 [34]
FSL J1-175	1/2b	Bergholz, 2010 [35]
FSL J1-208	4a	Roberts, 2006 [36]
FSL C1-115	3a	Fugett, 2006 [37]
FSL J1-094	1/2c	Fugett, 2006 [37]
FSL F2-695	4a	Roberts, 2006 [36]
FSL F2-501	4b	Roberts, 2006 [36]
FSL J2-071	4c	Roberts, 2006 [36]
FSL W1-110	4b	De Jesus and Whiting, 2003 [38]
FSL J1-158	4b	De Jesus and Whiting, 2003 [38]
FSL J1-169	3b	Fugett, 2006 [37]
FSL J1-049	3c	Fugett, 2006 [37]
FSL D4-0014	1/2a	Denes, 2015 [15]
FSL D4-0119	3	Denes, 2015 [15]
FSL R9-0915	7	Denes, 2015 [15]

### 2.2. Transmission Electron Microscopy

A purified, high titer phage sample (~1 × 10^10^ PFU/mL) was prepared for transmission electron microscopy (TEM) as previously described with modifications [26]. One milliliter of each phage sample was washed using a 0.1 M ammonium acetate solution (pH 7) and centrifuged at 21,000× *g* with a microcentrifuge (Thermo Fischer Scientific, Waltham, MA, USA). One drop of the phage sample was deposited onto a 150–200 mesh carbon-coated Formvar film copper grid (Electron Microscopy Sciences, Hatfield, PA, USA) and stained using 1% phosphotungstic acid (PTA; pH 7.4). Samples were imaged using a JEOL 1400 Flash transmission electron microscope at 120 kV. Images were analyzed using Fiji 3 v.2.0.0-rc-69/1.52p.

### 2.3. DNA Extraction and Genomic Analysis

Phage DNA were extracted by the phenol-chloroform method as previously described [26].

Libraries were prepared using a Nextera kit (Illumina, San Diego, CA, USA) and sequenced with an Illumina NextSeq 550 using 150 bp paired-end read chemistry. LP-027 was additionally long-read sequenced; the library was prepared using a Rapid Barcoding Kit (SQK-RBK0004; Oxford Nanopore Technologies, Oxford, UK) and sequenced with a MinIon. Illumina reads were trimmed using Trimmomatic (v0.35) [42] and read quality statistics were generated using FastQC (v0.11.7). Reads were mapped to the *L. monocytogenes* propagation host strain (F2365) genome in order to filter out host contamination reads. Assemblies were generated using SPAdes (v3.12.0) [43] and a hybrid assembly was generated with Unicycler (v0.4.8-beta) [44] for LP-027 using both Illumina and Nanopore reads. For some genomes, reads were subsampled to obtain better assemblies. Final assemblies were re-oriented to start at the large terminase subunit and annotated using RASTtk [45], with the pipeline modified to run “annotate-proteins-phage” before “annotate-proteins-kmer-v2.” Assembly statistics were generated using Quast (v4.6.3) [46], BBMap (v38.08) [47], and SAMtools (v1.8) [48]. Average nucleotide identity (ANI) values between phages from this study and those described previously by Denes et al. [41] were calculated using BLAST with JSpeciesWS [49]. Genome similarity maps of the representative phages and most similar previously described phages were created using EasyFig (v2.2.2) [50] with similarity calculated using both BLASTn and tBLASTx (v2.11.0+) [51]. The phage lifestyles were classified using PHACTS [52] and were additionally assessed by manually inspecting the genomes for genes related to lifestyle (e.g., integrases) and the phage genomes were used as queries to BLAST against the nr/nt database limited to *L. monocytogenes* (txid: 1639). Sequencing data and assemblies are available on NCBI under BioProject PRJNA688926.

### 2.4. Efficiencies of Plaquing and Relative Phage Activity

All ten *Listeria* phages and sixteen *L. monocytogenes* strains (except MACK) listed in Table 1 were used to conduct efficiency of plaquing (EOP) and relative phage activity (RPA) assays as previously described [25,53]. In brief, bacterial lawns were prepared with the double agar overly method and allowed to solidify. All the phages were amplified from the original phage stock and diluted to 1 × 10^7^ PFU/mL as a working stock. Serial dilutions were made from each working stock and spotted onto bacterial lawns. The EOP of each phage was determined from the highest dilution with countable plaques against the strain in question compared to the number of plaques against the phage propagation host strain. Similarly, the RPA of each phage was determined from the highest dilution with observable inhibitory activity against the strain in question compared to the phage propagation host strain. Inhibitory activity is defined as an observable inhibition of the growth of the bacterial lawn where the phage dilution was spotted either with or without the formation of any phage plaques. Three biological replicates were performed. EOP and RPA clustered heatmaps were generated using *pheatmap* in R [54].

### 2.5. One-Step Growth Curve

An exponential-phase culture of F2365 was infected with LP-020, LP-027, or LP-094 at a multiplicity of infection (MOI) of 0.1. The infected culture was incubated at 25 °C and 160 RPM for 3 h. To measure infected host cells and unabsorbed viable phages, two samples were taken at each time point. One sample was serially diluted and enumerated by the spot assay method immediately after collection, the other sample was treated with 5%(*v*/*v*) chloroform for 15 min and then serially diluted and enumerated by the spot assay method, and the plates were incubated at 25 °C for 12 h. Three biological replicates were performed.

### 2.6. Inhibition Growth Curve of Listeria monocytogenes F2365 by LP-020, LP-027, and LP-094

A measure of 2 mL of *L. monocytogenes* F2365 overnight culture was added into 100 mL LB-MOPS with 0.1% glucose, 1 mM CaCl_2_, and 1 mM MgCl_2_. The culture was incubated at 25 °C and 160 rpm until the OD_600nm_ grew to ~0.1. The culture was then diluted 10-fold with fresh supplemented LB-MOPS. A measure of 7 mL of the diluted culture was added to twelve sterile 15 mL glass culture tubes. Each tube was infected with LP-020, LP-027, or LP-094 at MOI = 0.1, 1, 10 with SM buffer as a negative control and incubated at 25 °C and 160 RPM for 15 h. The OD_600nm_ of each tube was measured every half an hour for 15 h on a Genesys 30 Visible spectrophotometer (Thermo-Fisher Scientific, Waltham, MA, USA). Three biological replicates were performed.

### 2.7. Inhibition Growth Curve of Listeria monocytogenes Cocktail by LP-020 and LP-094

Seven strains were used in this experiment: F2365 (4b), FSL J1-208 (4a), FSL F2-695 (4a), FSL F2-501 (4b), FSL J2-071 (4c), FSL W1-110 (4b), and FSL J1-148 (4b). Each strain was incubated in LB-MOPS supplemented with 0.1% glucose, 1 mM CaCl_2,_ and 1 mM MgCl_2_ at 25 °C and 160 rpm until the OD_600nm_ grew to ~0.1. A *L. monocytogenes* cocktail was prepared by transferring 1 mL of each strain into one 15 mL sterile tube and mixing by vortex mixer. The cocktail was then diluted 10-fold with fresh supplemented LB-MOPS. A total of 7 mL of the diluted cocktail was added to eight sterile 15 mL glass culture tubes. Each tube was infected with LP-020 or LP-094 with MOI = 0.1, 1, 10 or SM buffer as a negative control, grown at 25 °C, and shaken at 160 RPM for 15 h. The OD_600nm_ of each tube was measured every half an hour for 15 h. Three biological replicates were performed.

## 3. Results and Discussion

### 3.1. Transmission Electron Microscopy Imaging of Wild Type Listeria Phages Revealed Two Distinct Morphologies

**Figure 1:**
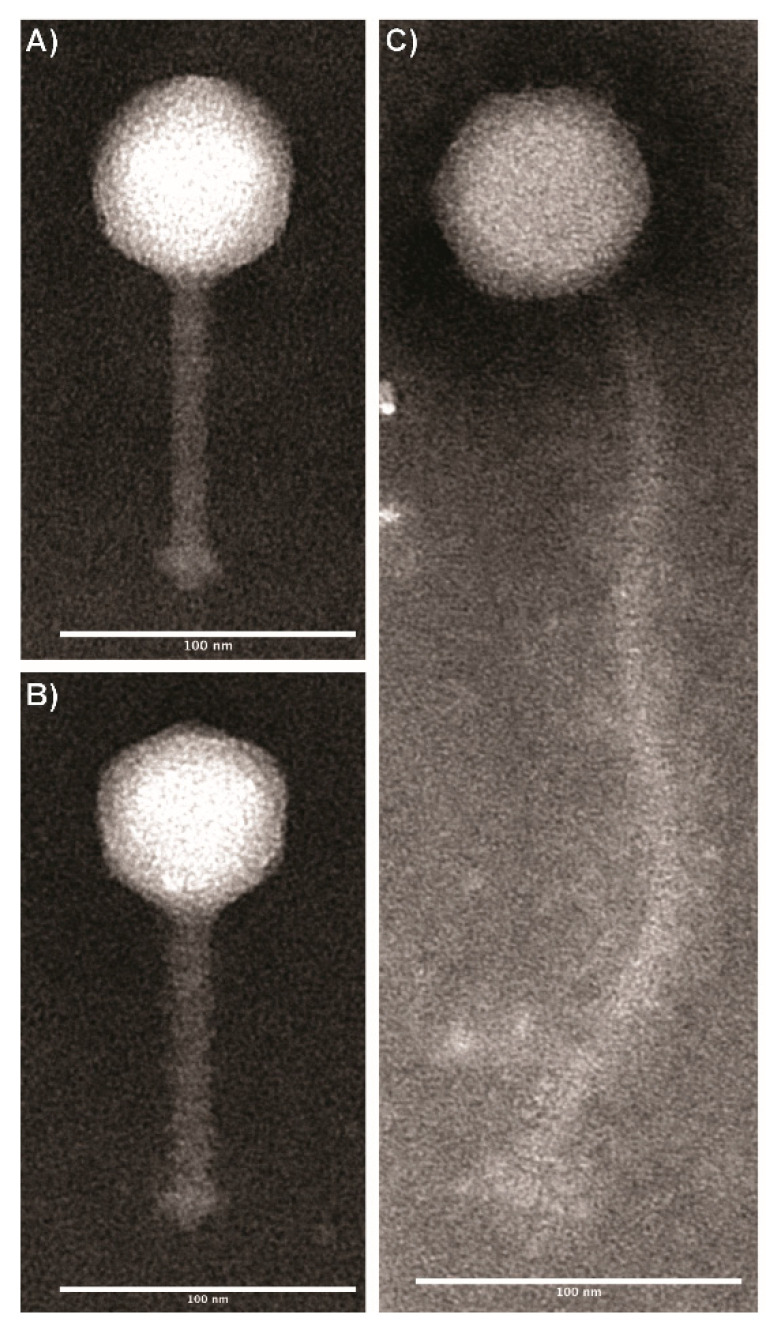
Transmission electron microscopy images of *Listeria* phages representing two morphologies: (**A**) LP-020 and (**B**) LP-094, characterized by an icosahedral capsid and a flexible, non-contractile tail; (**C**) LP-027, characterized by an icosahedral capsid and a long, flexible, non-contractile tail. Phages were stained with 1% phosphotungstic acid (pH &) and imaged at a final magnification of ×69,700–83,600. Images were analyzed using FIJI 3 (v2.0.0-rc-69/1.52p).

Based on morphological characteristics, all phages were classified within the *Siphoviridae* family (Figure 1). One group that includes two phages, LP-024 and LP-027, were found to have icosahedral capsids with flexible, elongated tails (Table 3). The second group also had icosahedral capsids; however, these phages were found to have short flexible tails (Table 3).

### 3.2. Genomic Analysis

**Figure 2:**
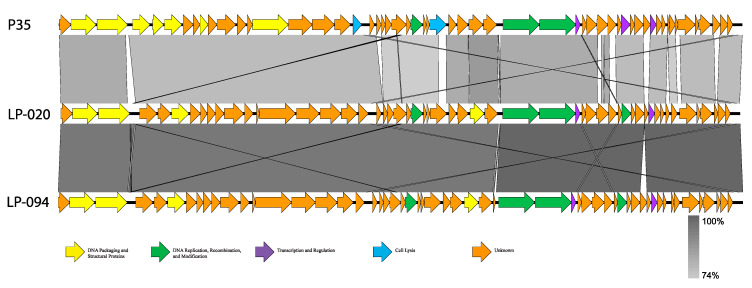
Linear BLASTn comparisons of representative P35-like *Listeria* phages. Genes are represented by arrows and are colored based on putative function (see key at bottom). The shaded region between genomes represents nucleotide similarity, with the darker gray representing higher similarity and lighter gray indicating lower similarity (see scale at bottom right).

All phage reads assembled into complete, single-contig genomes; genome statistics are presented in Table 4. Based on genome statistics, two distinct groups were evident. LP-024 and LP-027 were 41.0–41.4 kb, with G+C contents of 36.5–36.6%, and each contained 74 coding sequences (CDS) and no RNAs. The other phage genomes were 35.6–36.0 kb, with G+C contents of 39.9–40.0%, and each contained 54–57 CDS and no RNAs. The two distinct groups were also supported by average nucleotide identity (ANI) values (Table 5 and Table 6) and amino acid similarity (Figure S1). LP-024 and LP-027 had an ANI of 100.00% over 95.51–96.63% of their genomes and were most similar to LP-030-3 (99.99–100.00% ANI over 95.92–96.63% of their genomes) (Table 5), a putative temperate phage. LP-030-3 was previously classified as an Orthocluster IV siphovirus [41], with a 41.2 kb genome containing 73 predicted genes. Electron micrographs show that LP-030-3 has a long and rigid tail [41]. Morphology and genome features of LP-030-3 are consistent with the LP-027-like phages and are likely putative temperate phage [41]. These two phages are likely the same genus and species as LP-030-3, as they are above the 50% and 95% cutoffs for genus and species delineation. LP-030-3 is currently listed as a “unclassified Siphoviridae”, with no genus classification, on NCBI (NCBI:txid1458852) and is not included in the most recent ICTV Master Species List 2019.v1. The other phages had an ANIs of 97.66–100.00%, over 90.63–99.24% of their genomes, and were most similar to P35 (79.51–80.36% ANI over only 83.03–87.67% of their genomes) (Table 6, Figure 2), a lytic phage [41]. P35 was previously classified as an Orthocluster II siphovirus [41], with a 35.8 kb genome containing 56 predicted genes. Electron micrographs show that P35 has a short tail [41,55]. Morphology and genome features of P35 are consistent with the LP-020-like phages and they are likely putative obligate lytic phages [41,55]. Given the >50% nucleotide similarity to P35, this second group of phages likely belongs to the same genus [56]. However, they clearly qualify as a novel species, as they are well below the 95% similarity cutoff to be considered the same species as P35. P35 is currently listed as a “unclassified Siphoviridae,” with no genus classification, on NCBI (NCBI:txid330398) and is not included in the most recent ICTV Master Species List 2019.v1.

LP-024 and LP-027 were confidently predicted as having a temperate lifestyle by PHACTS (Table S1) and both genomes contained a phage integrase gene. This was further confirmed through BLAST, which showed they have high similarity to *L. monocytogenes* genomes (up to 99.79% identity and 86% query coverage). LP-020 and LP-057 were non-confidently predicted as having a lytic lifestyle and LP-021, LP-053, LP-054, LP-085, and LP-094 were non-confidently predicted as having a temperate lifestyle (Table S1). However, none of these genomes contained an integrase gene, and BLAST showed they did not have high similarity to any *L. monocytogenes* genomes (only up to 5% query coverage), indicating that they likely have a lytic lifestyle.

### 3.3. Host Range Analysis

**Figure 3:**
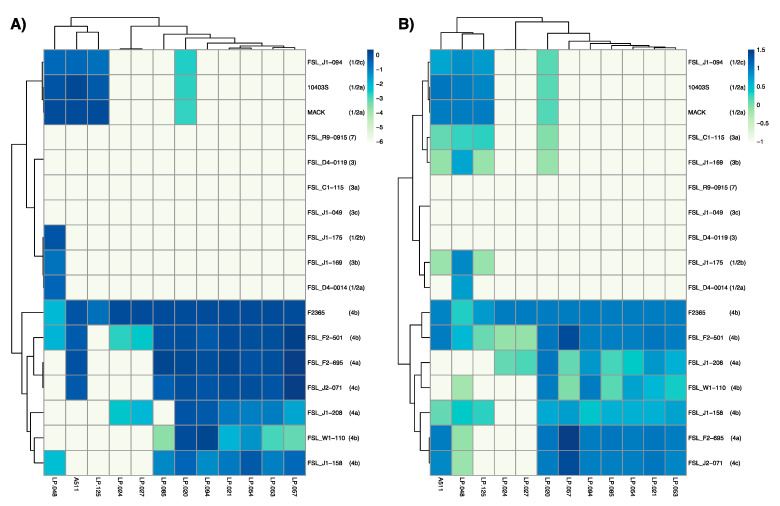
Host range analysis of *Listeria* phages against a panel of *Listeria monocytogenes* strains that represent different serotypes. Panel (**A**) represents efficiency of plaquing (EOP) results where values represent the log transformed efficiencies of plaquing of each phage against each bacterial strain compared to the phage propagation host strain. Panel (**B**) represents efficiency of activity (EOA) results where values represent the greatest dilution factor where phage activity was observed against each strain relative to the phage propagation host strain. Values are the mean of data from three biological replicates.

A511, which has been described as a broad host range phage [28], showed activity against 11 out of 17 *L. monocytogenes* (Figure 3A, Figure S2), but only formed visible plaques on seven strains (Figure 3B, Figure S2). Similarly, two previously characterized phages, LP-048 and LP-125, showed activity against a broad range of strains (thirteen and nine strains, respectively), compared to their ability to form plaques on the same strains (nine and four strains, respectively). All three of these *Pecentumvirus* phages were unable to infect all serotype 4b and 4a strains.

The phages selected for this study that showed strong activity against serotype 4b strain F2365 can be divided into two groups, and these groupings are consistent with those based on observed morphological characteristics. The first group, comprised of LP-024 and LP-027, showed a very narrow host range, forming plaques on F2364 (4b), FSL F2-501 (4b), and FSL J1-208 (4a). The second group, comprised of LP-020, LP-021, LP-053, LP-054, LP-057, LP-085, and LP-094, showed activity to all *L. monocytogenes* serotype 4 strains, and formed visible plaques on nearly all *L. monocytogenes* serotype 4 strains. Interestingly, LP-020 is the only phage in this group that was able to form plaques on non-serotype 4 strains.

FSL R9-0915 (7), FSL J1-049 (3c), and FSL D4-0119 (3) were the only three strains that were resistant to all phages in this study, although this is not surprising as these strains have consistently shown resistance to phage infection [17,25].

Based on the combination of their morphological characteristics and the results of their EOP and RPA, LP-020, LP-027, and LP-094 were selected as representative phages for further evaluation in this study.

### 3.4. One-Step Growth Curves

**Figure 4:**
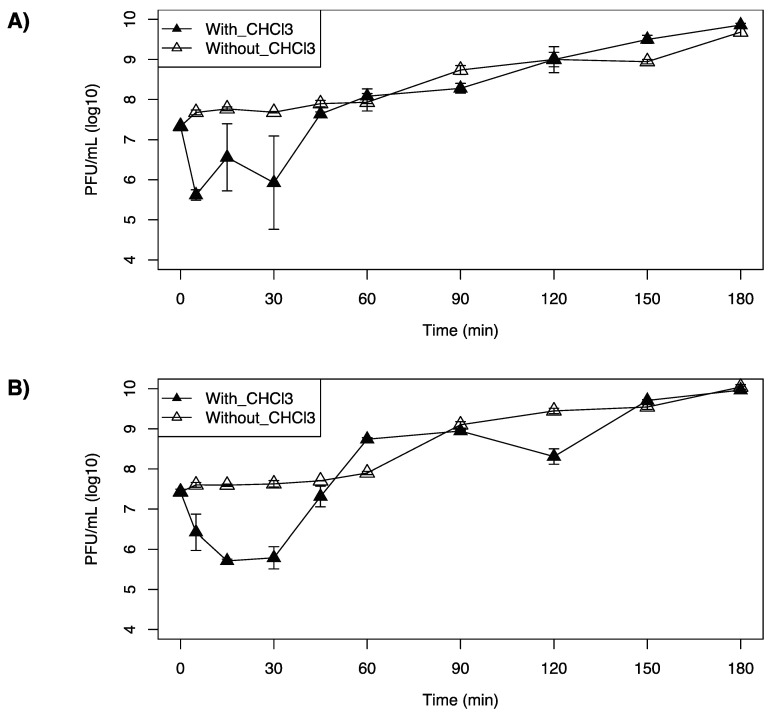

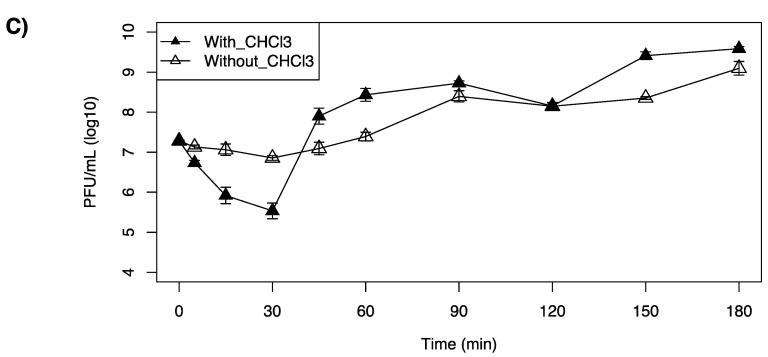
One-step growth curve of *Listeria monocytogenes* F2365 treated with (**A**) LP-20, (**B**) LP-0-27, or (**C**) LP-094 at a MOI = 0.1 at 25 °C. Filled triangles represent the phage titer in chloroform treated samples and unfilled triangles represent the phage titer in untreated samples. Data are mean values of three biological replicates and error bars represent standard error.

LP-020 was found to have a short adsorption time, in which 97.9% of LP-020 adsorbed to F2365 in 5 min. This is in contrast with LP-027 and LP-094, in which 30 min was required to attain adsorptions of 96.4% and 97.8%, respectively. One-step growth curve analysis of LP-020 showed a latent period of 60~90 min, an eclipse period of 5~15 min, and a burst size of ~9.7 (SE, 2.9) PFU/cell (Table 7, Figure 4A). One-step growth curve analysis of LP-027 showed a latent period of 45~60 min, an eclipse periods were 30~45 min, and a burst size of ~34.4 (SE, 5.8) PFU/cell (Table 7, Figure 4B). One-step growth curve analysis of LP-094 showed a latent period of 30~45 min, an eclipse period of 30~45 min, and a burst size of ~28.3 (SE, 4.1) PFU/cell (Table 7, Figure 4C).

However, the one-step growth curve of LP-020 showed a fluctuation in completed phage numbers between 5 min and 30 min (Figure 4A).

### 3.5. Inhibition Growth Curve of Listeria monocytogenes F2365 by LP-020, LP-027, and LP-094

**Figure 5:**
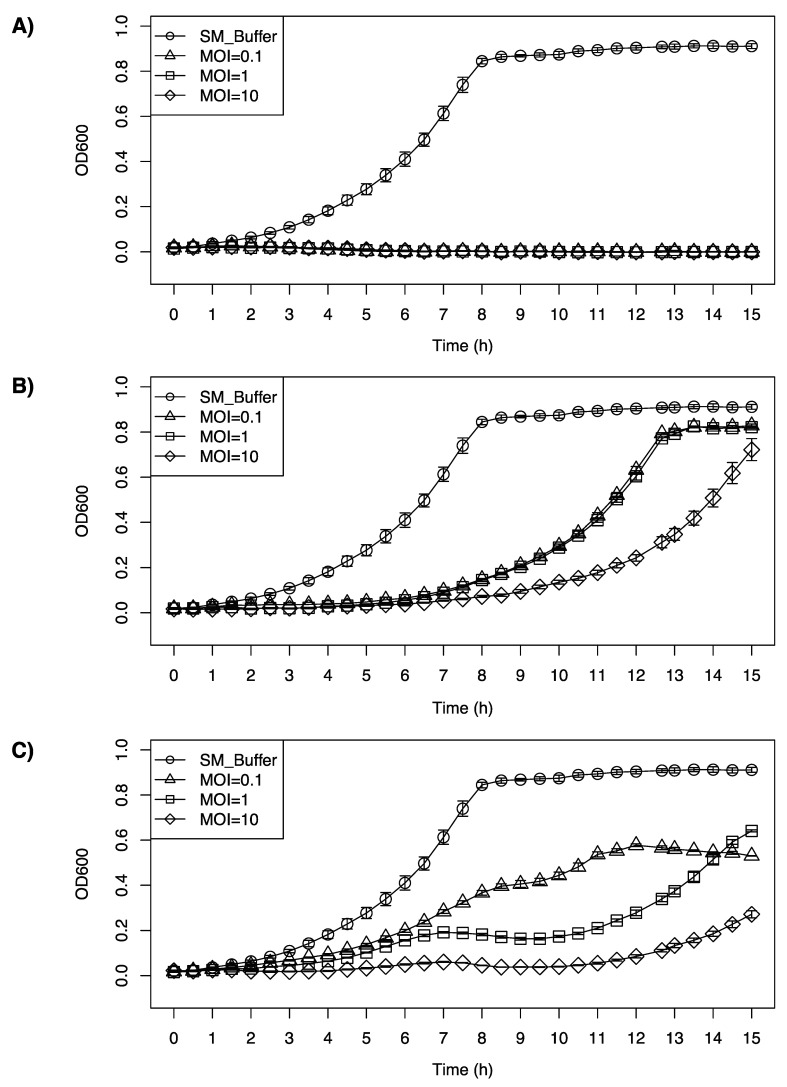
Inhibition growth curve of *Listeria monocytogenes* F2365 treated with (**A**) LP-020, (**B**) LP-027 or (**C**) LP-094 at different MOIs. Unfilled circles represent SM buffer control, unfilled triangles represent samples treated at an MOI of 0.1, unfilled squares represent samples treated at an MOI of 1, and diamonds represent samples treated at a MOI of 10. Data are mean values of three biological replicates and error bars represent the standard error.

All the phages tested were able to inhibit the growth of F2365, at a range of different multiplicities of infection (MOIs). LP-020 showed high efficiency in inhibiting the growth of F2365. Even with the lowest concentration (MOI = 0.1), LP-020 can keep the OD_600nm_ of F2365 under 0.03 for 15 h (Figure 5A). LP-027 could keep the OD_600nm_ of F2365 under 0.1 for 7 h with MOI = 0.1 and MOI = 1, and for 9 h with MOI = 10 (Figure 5B). LP-094 could keep the OD_600nm_ of the F2365 under 0.1 for 4 h and 4.5 h, with MOI = 0.1 and MOI = 1, respectively, and for 12 h with MOI = 10. Interestingly, after 12 h of incubation, the sample infected with LP-094 at MOI = 1 has a higher OD_600nm_ than the sample infected with LP-094 at MOI = 0.1, possibly due to the lower MOI (0.1) providing an opportunity for host to continue during early infection, leading to greater levels of phage production during later infection (Figure 5C).

### 3.6. Inhibition Growth Curve of Listeria monocytogenes Cocktail by LP-020 and LP-094

**Figure 6:**
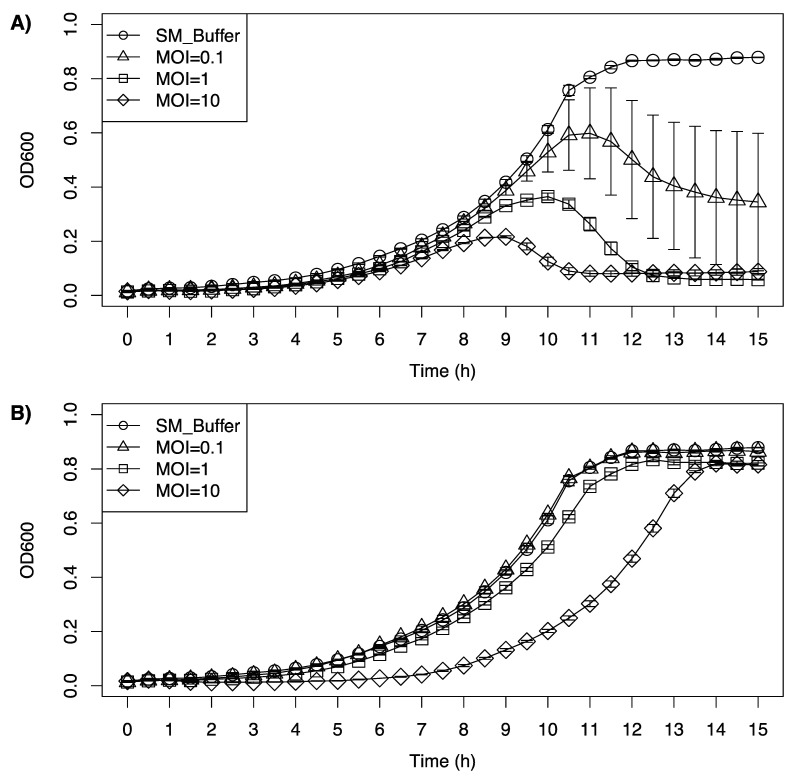
Inhibition growth curve of a cocktail of *Listeria monocytogenes* serotype 4 strains (F2365 (4b), FSL J1-208 (4a), FSL F2-695 (4a), FSL F2-501 (4b), FSL J2-071 (4c), FSL W1-110 (4b), and FSL J1-148 (4b)) treated with (**A**) LP-020 and (**B**) LP-094 at different MOIs. Unfilled circles represent SM buffer control, unfilled triangles represent samples treated at a MOI of 0.1, unfilled squares represent samples treated at a MOI of 1, and diamonds represent samples treated at a MOI of 10. Data are mean values of three biological replicates and error bars represent the standard error.

LP-020 was able to inhibit the growth of a cocktail of *L. monocytogenes* serotype 4 at all tested MOIs, while LP-094 only showed an inhibitory effect with the highest MOI (Figure 6). For the first 8 h after LP-020 incubation, the growth curve of the cocktail showed no substantial difference. The growth curve of the cocktail treated with different dosages of LP-020 reached its peak at 11 h, 10 h, and 9 h, respectively. After reaching their peak, the bacterial cocktails treated with LP-020 showed a reduction in OD_600nm_, with the 1 and 10 MOI infections reducing the OD_600nm_ to 0.06 and 0.08, respectively. Interestingly, the low MOI treatment group (MOI = 0.1) showed a large variation in OD_600__nm_, which may be due to the diversity of the host strains in the cocktail generating a more varied response to phage infection as the individual concentrations of each *L. monocytogenes* strain could vary from replicate to replicate due to complex growth interactions (Figure 6A). For the LP-094 infection, low MOI (MOI = 0.1, 1) showed no major influence of the growth curve of the cocktail. However, LP-094 can keep the OD_600nm_ of the cocktail under 0.1 for 8 h with a high dosage (MOI = 10) (Figure 6B). Compared with LP-094, LP-020′s ability to reduce the OD_600nm_ of a cocktail of serotype 4a, 4b, and 4c *Listeria* strains suggests it could be a promising phage for biocontrol applications in food processing environments.

## 4. Conclusions

In this study we describe two groups of *Listeria* phages that showed high levels of infectivity against serotype 4 strains of *L. monocytogenes*. One of these groups of phages, LP-020-like phages showed a level of nucleotide dissimilarity with previously sequenced phages that is well above the 5% cutoff for qualifying as a novel species. Inhibition assays of *L. monocytogenes* against a cocktail of serotype 4 strains (4a, 4b, and 4c) confirmed that EOP and RPA assays were predictive of a phage’s inhibitory potential. We identified LP-020 as a phage that may be particularly useful in biocontrol settings. It shows strong activity against all serotype 4a, 4b, and 4c strains tested here. Given that some types of phage resistance is specific to a single species or closely related group of phages, such as CRISPR-Cas mediated resistance, it is critical to consider using diverse cocktails of phages. LP-020 may serve together with *Listeria* phages from other groups, such as *Pecentumviruses* [11,57,58] and *Homburgviruses* [26,59,60], to increase both the diversity of *Listeria* phage cocktails and their infectivity against the problematic serotype 4 strains that are often associated with human illness. Further, the temperate phages described here, which are similar to LP-030-3, may be useful as biocontrol agents if their integrases are knocked out, which has previously been shown for the temperate *Listeria* phage PSA [61].

## Figures and Tables

**Table 2:** *Listeria monocytogenes* phages.

Phages	Description	Reference or Original
A511		Loessner and Busse, 1990 [39]; Klumpp, Jochen, et al., 2008 [40]
LP-020		Vongkamjan et al., 2012
LP-021		Vongkamjan et al., 2012 [17]
LP-024		Vongkamjan et al., 2012 [17]
LP-027		Vongkamjan et al., 2012 [17]
LP-048	P100-like phage	Vongkamjan et al., 2012; Denes et al., 2014 [17,41]
LP-053		Vongkamjan et al., 2012 [17,41]
LP-054		Vongkamjan et al., 2012 [17]
LP-057		Vongkamjan et al., 2012 [17]
LP-085		Vongkamjan et al., 2012 [17]
LP-094		Vongkamjan et al., 2012 [17]
LP-125	P100-like phage	Vongkamjan et al., 2012; Denes et al., 2014 [17,41]

**Table 3:** Morphology of *Listeria monocytogenes* phages.

*Listeria monocytogenes* Phages	Capsid Diameter (nm)	Tail Length (nm)	Tail Width (nm)
LP-020	73.40 ± 0.61	104.80 ± 1.45	11.54 ± 0.09
LP-021	69.92 ± 4.62	102.45 ± 2.32	11.01 ± 0.61
LP-024	66.10 ± 5.07	291.29 ± 18.56	8.25 ± 2.54
LP-027	70.35 ± 2.40	295.38 ± 10.42	7.95 ± 2.09
LP-053	68.03 ± 4.76	104.01 ± 3.00	11.88 ± 1.91
LP-054	73.70 ± 0.50	100.71 ± 3.55	11.43 ± 0.33
LP-057	73.60 ± 0.37	102.45 ± 1.62	13.46 ± 0.90
LP-085	72.22 ± 0.62	104.02 ± 3.19	12.61 ± 0.77
LP-094	71.75 ± 2.98	100.49 ± 2.87	12.81 ± 0.97

**Table 4:** Assembly statistics for *Listeria* phages.

Phage	BioSample ID	Length (bp)	Avg. Illumina Read Coverage (X)	G+C (%)	No. CDS	No. RNAs
LP-020	SAMN17217625	35,609	326.6	40.0	54	0
LP-021	SAMN17217626	35,610	344.1	40.0	54	0
LP-024	SAMN17217627	40,964	153.7	36.5	74	0
LP-027	SAMN17217628	41,120	89.6	36.6	74	0
LP-053	SAMN17217629	35,951	143.5	40.0	57	0
LP-054	SAMN17217630	35,951	524.9	40.0	57	0
LP-057	SAMN17217631	35,608	1001.1	40.0	54	0
LP-085	SAMN17217632	35,951	650.4	39.9	57	0
LP-094	SAMN17217633	35,885	3397.6	40.0	56	0

**Table 5:** JSpecies results for LP-030-03-like *Listeria* phages.

	Average Nucleotide Identity (ANI; %) [Aligned Nucleotides (%)]					
Phage	LP-024	LP-027	LP-030-3	A500	A118	A006
**LP-024**		100.00 [96.63]	100.00 [96.63]	92.81 [63.99]	86.34 [52.10]	92.00 [6.82]
**LP-027**	100.00 [95.51]		100.00 [95.92]	92.77 [63.41]	86.10 [50.80]	91.76 [5.70]
**LP-030-3**	99.99 [96.21]	100.00 [96.57]		92.07 [62.93]	84.54 [47.14]	88.41 [6.80]
**A500**	92.74 [68.96]	92.74 [68.96]	92.74 [69.01]		84.52 [49.43]	89.93 [6.42]
**A118**	84.79 [53.25]	84.78 [53.48]	84.78 [53.51]	84.00 [47.63]		95.38 [23.01]
**A006**	89.85 [7.30]	89.54 [9.18]	89.54 [9.18]	84.93 [4.69]	94.91 [27.18]	

**Table 6:** JSpecies results for P35-like *Listeria* phages.

	Average Nucleotide Identity (ANI; %) [Aligned Nucleotides (%)]								
Phage	LP-020	LP-021	LP-053	LP-054	LP-057	LP-085	LP-094	P35	P40
**LP-020**		99.99 [97.36]	97.68 [94.41]	97.68 [94.41]	98.71 [97.35]	97.86 [94.41]	97.66 [93.39]	79.51 [85.61]	61.76 [32.56]
**LP-021**	99.99 [97.36]		97.69 [94.41]	97.69 [94.41]	98.72 [97.34]	97.87 [94.41]	97.68 [93.39]	79.52 [85.63]	61.76 [32.55]
**LP-053**	97.77 [90.63]	97.78 [90.63]		100.00 [99.24]	99.15 [90.64]	99.74 [99.24]	99.98 [98.21]	79.85 [87.67]	61.47 [33.98]
**LP-054**	97.77 [90.63]	97.78 [90.63]	100.00 [99.24]		99.15 [90.64]	99.74 [99.24]	99.98 [98.21]	79.85 [87.67]	61.47 [33.98]
**LP-057**	98.71 [97.34]	98.72 [97.35]	98.97 [94.42]	98.97 [94.42]		99.02 [94.42]	98.96 [93.40]	79.62 [85.79]	61.80 [32.41]
**LP-085**	97.97 [90.63]	97.98 [90.63]	99.74 [99.24]	99.74 [99.24]	99.22 [90.64]		99.73 [98.21]	79.87 [87.67]	61.51 [33.94]
**LP-094**	97.75 [91.58]	97.76 [91.58]	99.98 [98.24]	99.98 [98.24]	99.08 [91.59]	99.73 [98.24]		79.98 [86.27]	61.54 [32.97]
**P35**	79.85 [83.06]	79.86 [83.07]	80.35 [84.12]	80.35 [84.12]	80.02 [83.03]	80.36 [84.12]	80.33 [84.12]		63.10 [23.90]
**P40**	61.52 [25.48]	61.81 [24.84]	62.26 [22.30]	62.26 [22.30]	62.37 [22.14]	62.36 [22.26]	62.29 [22.30]	62.80 [24.06]	

**Table 7:** Infection kinetics summary.

*Listeria monocytogenes* Phages	LP-020	LP-027	LP-094
**Adsorption Time(min)**	5	30	30
**Adsorption Rate(%)**	97.9 ± 0.5	96.4 ± 2.3	97.8 ± 1.0
**Latent Period(min)**	60~90	45~60	30~45
**Eclipse Period(min)**	5~15	30~45	30~45
**Burst Size(PFU/cell)**	9.7 ± 2.9	34.4 ± 5.8	28.3 ± 4.1

## Data Availability

Sequencing data and assemblies are available on NCBI under BioProject PRJNA688926.

## Supplementary Materials

Supplementary materials can be found at https://www.mdpi.com/article/10.3390/v13040671/s1.
