# Supplementary material for: Characterization of a Novel Group of Listeria Phages That Target Serotype 4b Listeria monocytogenes
Source: Viruses. 2021 Apr 14;13(4):671. doi: 10.3390/v13040671 (PMC8070769; doi:10.3390/v13040671)
Supplement: Supplementary file 1 [file viruses-13-00671-s001.pdf]

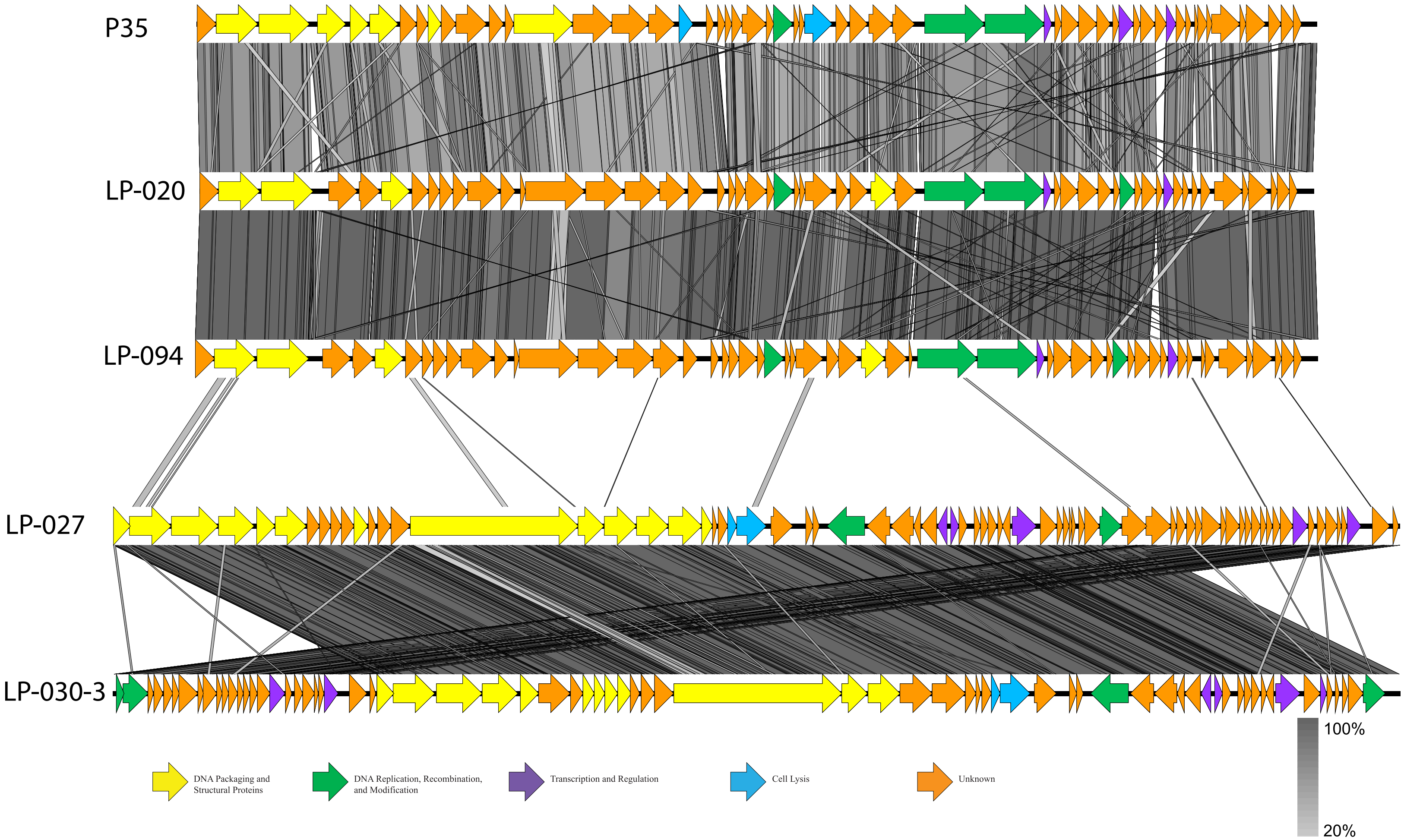

Figure S1. Linear tBLASTx comparrisons of representative P35-like and LP-30-3-like Listeria phages. Genes are represented by arrows and are colored based on putative function (see key at bottom). The shaded region between genomes represents amino acid similarity, with darker gray representing higher similarity and lighter gray indicating lower similarity (see scale at bottom right).

Table S1. Phage lifestyle prediction.

Phage	Predicted Class	Prediction Confidence	Averaged Probability	Standard Deviation
LP-024	Temperate	Confident	0.592	0.038
LP-027	Temperate	Confident	0.589	0.032
LP-020	Lytic	Non-Confident	0.507	0.03
LP-021	Temperate	Non-Confident	0.512	0.036
LP-053	Temperate	Non-Confident	0.512	0.057
LP-054	Temperate	Non-Confident	0.514	0.045
LP-057	Lytic	Non-Confident	0.507	0.024
LP-085	Temperate	Non-Confident	0.531	0.035
LP-094	Temperate	Non-Confident	0.511	0.039

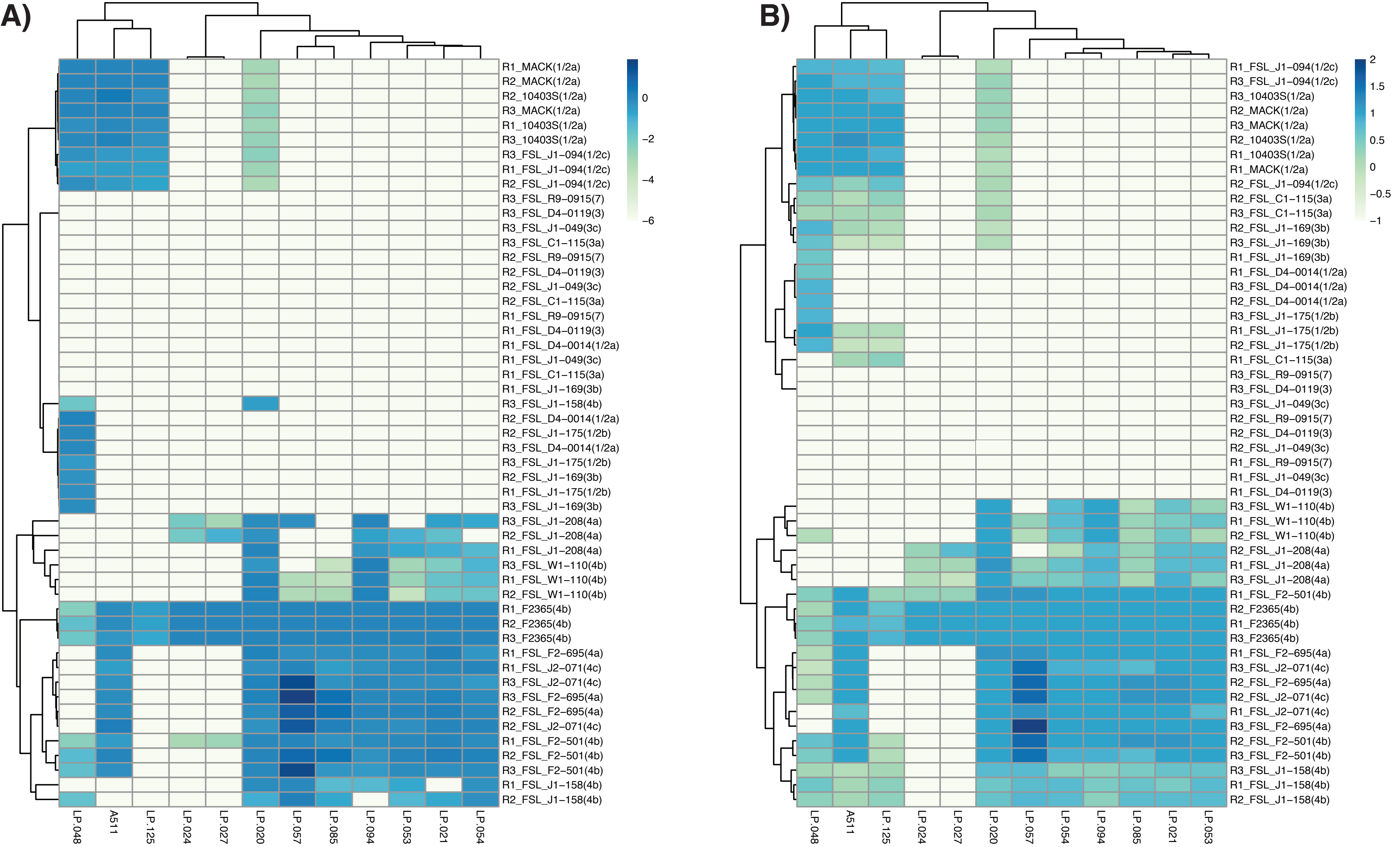

Figure S2. Host range analysis of Listeria phages against a panel of Listeria monocytogenes strains that represent different serotypes. Panel **A)** represent the clustered efficiency of plaquing (EOP) results of three individual replicates where values represent the log transformed efficiencies of plaquing of each phage against each bacterial strain compared to the phage propagation host strain.. Panel **B)** values represent the clustered efficiency of activity (EOA) results of three individual replicates where values represent the greatest dilution factor where inhibitory phage activity was observed against each strain relative to the phage propagation host strain.
